# Supplementary material for: Eigenstate control of plasmon wavepackets with electron-channel blockade
Source: Nat Commun. 2025 Nov 12;16:9942. doi: 10.1038/s41467-025-64876-z (PMC12612246; doi:10.1038/s41467-025-64876-z)
Supplement: Supplementary file 1 — Supplementary Information [file 41467_2025_64876_MOESM1_ESM.pdf]

Supplementary Information
Eigenstate control of plasmon wavepackets with electron-channel blockade

Shintaro Takada[1,2,3,4,⋆], Giorgos Georgiou[5], Junliang Wang[6], Yuma Okazaki[1], Shuji Nakamura[1], David Pomaranski[7], Arne Ludwig[8], Andreas D. Wieck[8], Michihisa Yamamoto[7,9], Christopher Bäuerle[6], & Nobu-Hisa Kaneko[1]

[1] National Institute of Advanced Industrial Science and Technology (AIST), National Metrology Institute of Japan (NMIJ), 1-1-1 Umezono, Tsukuba, Ibaraki 305-8563, Japan

[2] Department of Physics, Graduate School of Science, University of Osaka, Toyonaka, 560-0043, Japan

[3] Institute for Open and Transdisciplinary Research Initiatives, University of Osaka, Suita, 560-8531, Japan

[4] Center for Quantum Information and Quantum Biology (QIQB), University of Osaka, Osaka 565-0871, Japan

[5] James Watt School of Engineering, Electronics and Nanoscale Engineering, University of Glasgow, Glasgow, G12 8QQ, U.K.

[6] Université Grenoble Alpes, CNRS, Grenoble INP, Institut Néel, 38000 Grenoble, France

[7] Department of Applied Physics, University of Tokyo, Bunkyo-ku, Tokyo, 113-8656, Japan

[8] Lehrstuhl für Angewandte Festkörperphysik, Ruhr-Universität Bochum Universitätsstraße 150, D-44780 Bochum, Germany

[9] Center for Emergent Matter Science, RIKEN, Wako, Saitama, 351-0198, Japan

⋆ corresponding author: takada@phys.sci.osaka-u.ac.jp

1 Supplementary Notes

Supplementary Note 1: Analogy to IMI - SPP Modes

A potential analogy between the plasmon eigenstates in a quasi-one-dimensional quantum wire and the surface plasmon polariton (SPP) modes in insulator-metal-insulator (IMI) structures is discussed here. While both systems exhibit mode modulation dependent on the width of the central conducting regions, several distinctions are crucital:

- **Dispersion Relation:** The plasmon mode in our system exhibits a linear dispersion, in contrast to the more complex, quadratic-like dispersion of SPPs in IMI structures.
- **Mode Multiplicity:** A simple IMI structure supports only two SPP branches (high- and low-energy). In our system, with N non-interacting conduction channels (or $2N$ including spin), we obtain N spin modes and N charge modes. Among the charge modes, one special mode—the plasmon mode—dominates charge transport. When a wavepacket is excited via a voltage pulse at an Ohmic contact, it primarily couples to this plasmon mode.
- **Opposite Tendency in Splitting:** In IMI structures, the energy splitting increases as the middle metal layer becomes thinner. In contrast, in our system, a wider quantum wire supports more conduction channels, leading to stronger Coulomb interactions and a faster plasmon mode. The other charge modes

remain near charge neutrality and propagate close to the Fermi velocity, resulting in a larger energy separation for wider wires—an opposite trend.

- **Alternative Analogy:** The energy splitting between non-interacting conduction channels indeed increases as the wire becomes narrower (see Supplementary Figure 6 of Ref. 31 of the main text), which may offer a closer analogy to IMI-SPP splitting. However, our focus is on the plasmon mode arising from Coulomb interactions between these channels, which is fundamentally different from the electromagnetic origin of SPPs.

2 Supplementary Figures

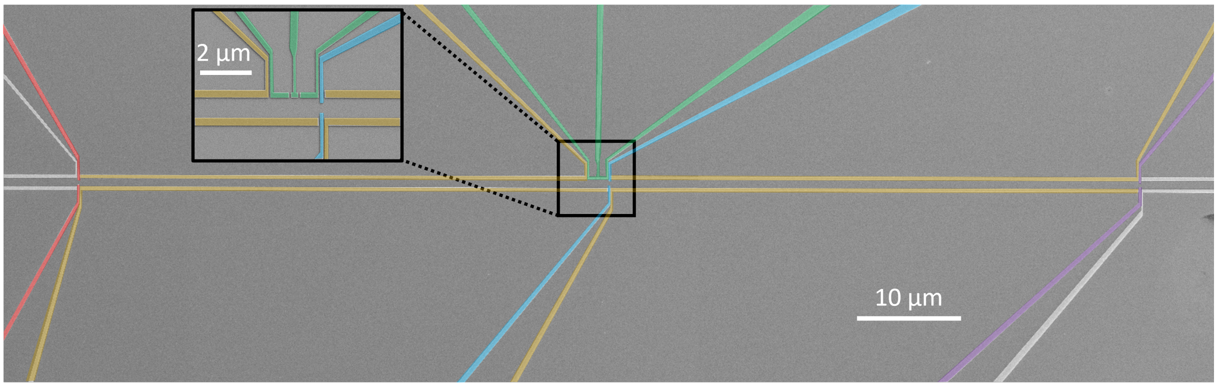

Supplementary Fig. 1: Scanning electron micrograph of the device. False colours are used to indicate the gates used for the experiment and correspond to the ones employed in Fig. 1a. The inset is the focus around QPC2. The gates without the false colours are not used in this experiment.

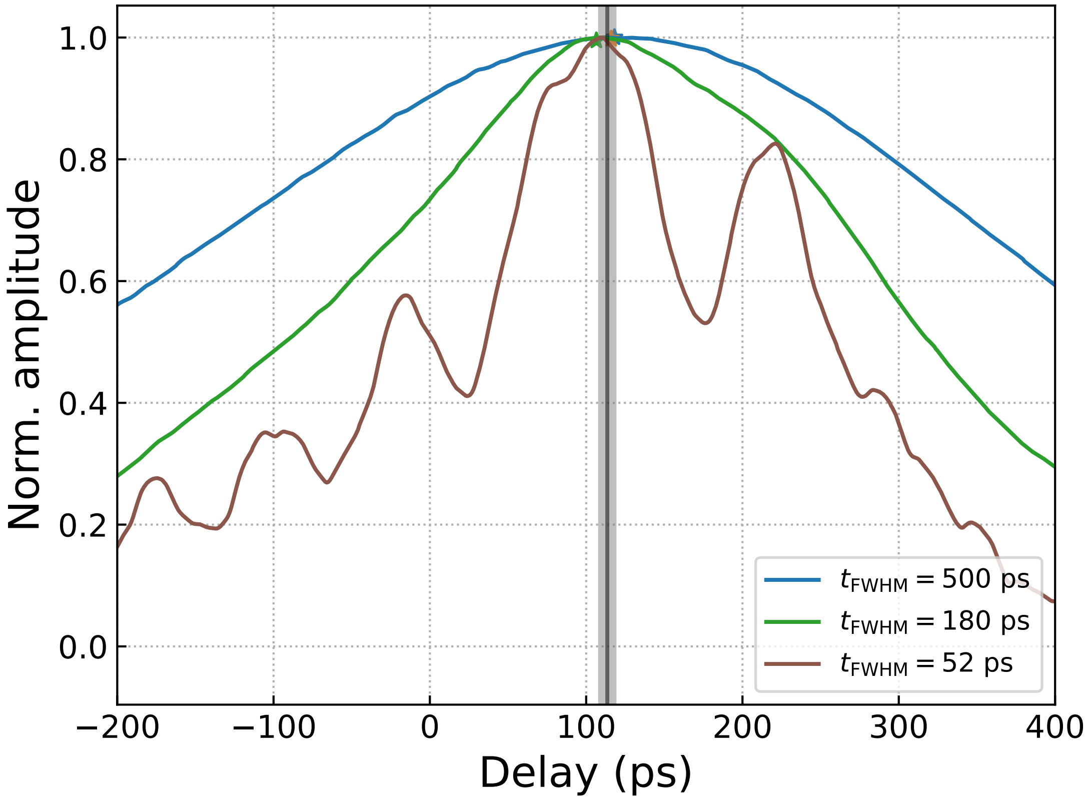

Supplementary Fig. 2: Calibration of RF line delay time using a two dimensional plasmon. A two dimensional plasmon is excited by applying the voltage pulse with different full width at half maximum (FWHM) on the contact, O_{i} without applying any voltage on the gates (w1, w2, QPC1, QPC2, g_{res}). The time-resolved measurement is performed by using QPC3 as explained in the main text. The peak position does not change for different pulse lengths and is estimated to be 112 ± 7 ps.

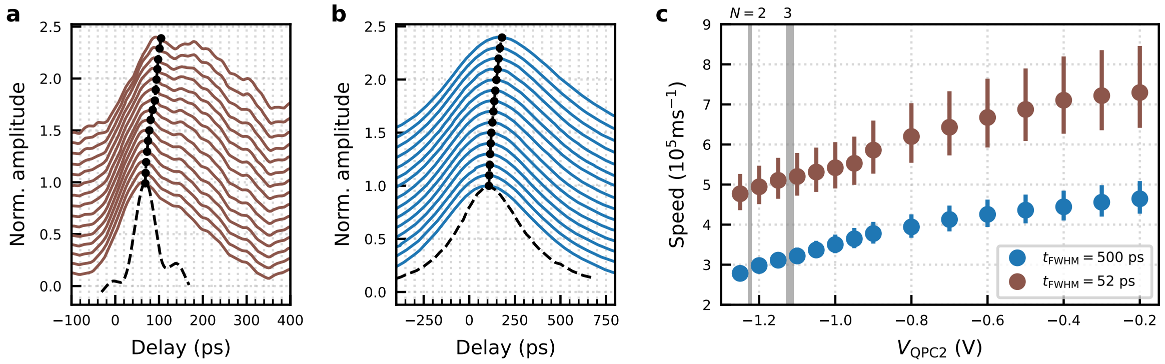

Supplementary Fig. 3: Local control of the number of transmitting electron conduction channels in $50\,\mu$m-**quantum wire. a, b.** Time-resolved measurement of plasmon wavepackets excited by 52 ps-long voltage pulse (**a**) and 500 ps-long voltage pulse (**b**) for different voltages on the gates of QPC2. The amplitude is normalised to one. Each curve is offset vertically for clarity. The gate voltage V_{QPC2} was stepped from $-0.2\,\mathrm{V}$ at the bottom to $-1.25\,\mathrm{V}$ at the top. The gate voltage V_{w2} was fixed to $-0.7\,\mathrm{V}$. The peak position is indicated by the black circles. The shape of the voltage pulse used to excite the plasmon wavepacket is drawn by the black dashed line. **c.** Speed of the plasmon wavepackets calculated from the peak delay indicated by the black circles in **a, b**. Here the number N on top of the grey shaded gate voltage indicates the number of transmitting electron channels across QPC2, which is determined by the observation of the quantised conductance. The data in **a** is in contrast to the data in Fig. 2a in 100 µm quantum wire. For this 50 µm quantum wire case, the wavepacket length is neither much smaller nor larger than double the cavity length ($L_{\mathrm{p}} \sim 2L$), representing an intermediate, or boundary, situation. The result is a shift in the peak delay as a function of QPC2 voltage, but the main peak is followed by several sub-peaks. This suggests that the wavepacket is not in a well-defined eigenstate and spreads into several different charge modes after passing through QPC2.

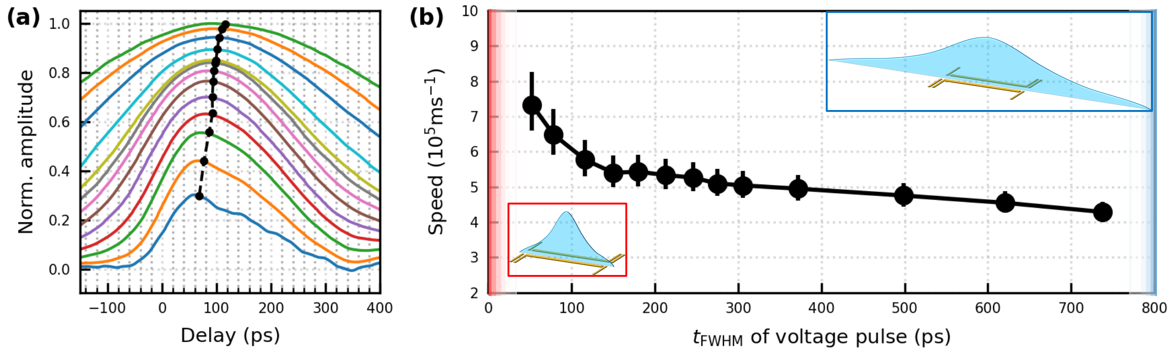

Supplementary Fig. 4: Temporal width dependent plasmon speed in 50 µm-**quantum wire. a.** Time-resolved measurement of plasmon wavepackets excited by the voltage pulse having different t_{FWHM}. t_{FWHM} of each pulse is as follows: 52 ps (bottom blue curve), 78 ps, 116 ps, 150 ps, 180 ps, 213 ps, 246 ps, 275 ps, 306 ps, 372 ps, 500 ps, 631 ps, and 737 ps. In this measurement, V_{QPC2} is fixed to -0.2 V and V_{w2} is fixed to -0.7 V. The peak position detected by Lorentzian curve fitting is indicated by the black points. **b.** Speed of plasmon wavepackets as a function of FHWM of the voltage pulse used for excitation. Schematics inside the figure show the situation where $L_{\mathrm{p}} \lesssim L$ (red box) and $L_{\mathrm{p}} > L$ (blue box). For longer plasmon pulses, the plasmon speed gradually decreases, as also shown in Fig. 2. In the adiabatic limit, where the plasmon temporal duration is large and approaches the DC transport limit, the plasmon speed is decreased and converges towards the electron's Fermi velocity in the 2DEG, $\sim 2.3 \times 10^5$ m/s. At this limit, the plasmon speed does not depend much on the plasmon duration. In contrast, by decreasing the plasmon duration, we observe that the speed is linearly increased. In the non-adiabatic limit, where the plasmon duration is shorter than the quantum wire, we observe a rapid increase of the plasmon speed ($<$ 100 ps). This suggests that the interactions between the electrons becomes important, thus forcing the plasmon to spread into other conduction channels and consequently the speed to be renormalised. In this limit, there is one dominant fast plasmon speed.

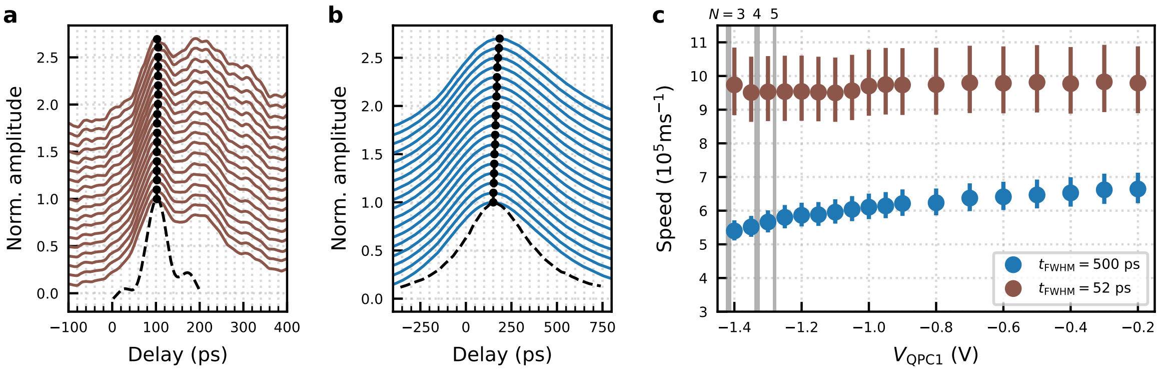

Supplementary Fig. 5: Local control of the number of transmitting electron channels in $100\,\mu$m**-quantum wire at** $4\,$K**. a, b.** Time-resolved measurement of plasmon wavepackets excited by $52\,$ps-long voltage pulse (**a**) and $500\,$ps-long voltage pulse (**b**) for different voltages on the gates of QPC1. The amplitude is normalised to one. Each curve is offset vertically for clarity. The gate voltage V_{QPC1} was stepped from $-0.2\,$V at the bottom to $-1.4\,$V at the top. The gate voltage $V_{\mathrm{w1,w2}}$ was fixed to $-0.7\,$V. The peak position is indicated by the black circles. The shape of the voltage pulse used to excite the plasmon wavepacket is drawn by the black dashed line. **c.** Speed of the plasmon wavepackets calculated from the peak delay indicated by the black circles in **a, b**. Here the number N on top of the grey shaded gate voltage indicates the number of transmitting electron channels across QPC1, which is determined by the observation of the quantised conductance. The gate voltage configuration is slightly modified from the same measurement at the base temperature shown in Fig. 2. This results in the slightly different absolute speeds of plasmon wavepackets.

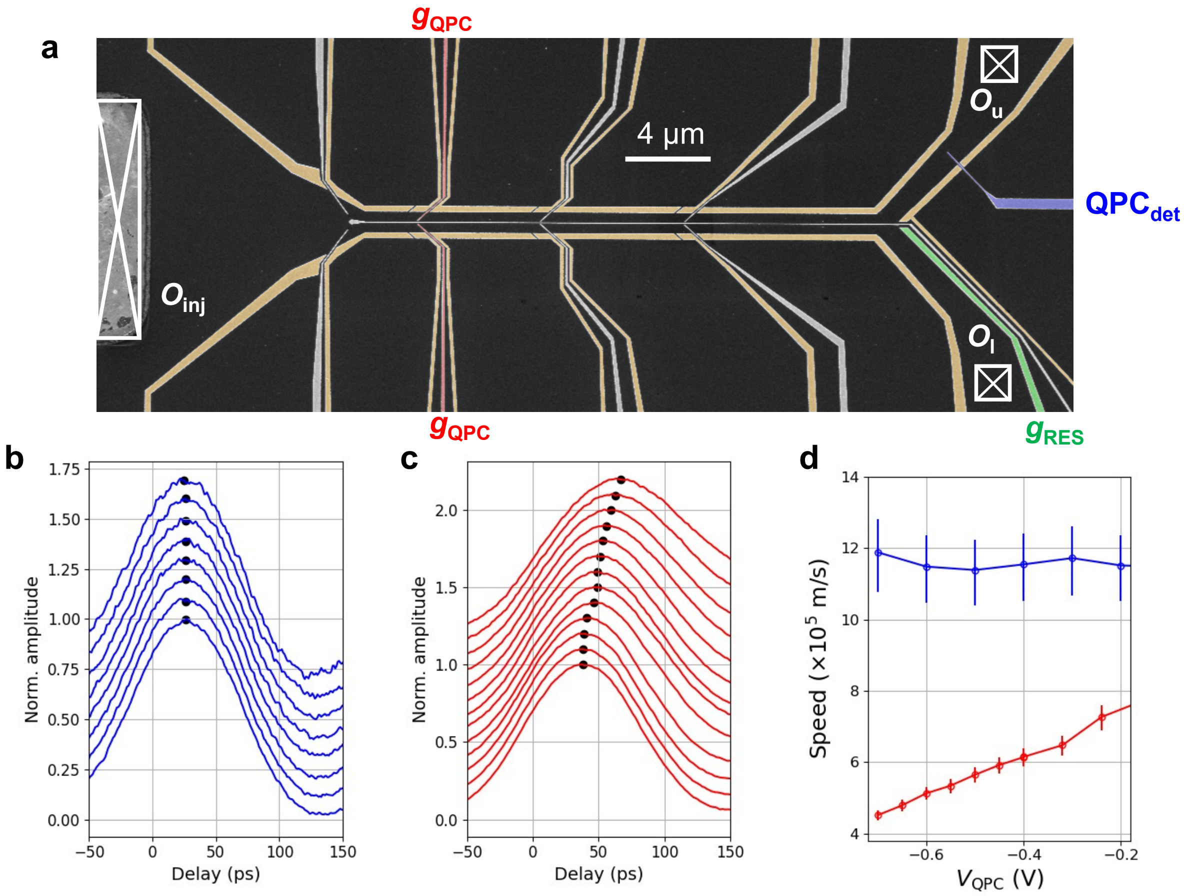

Supplementary Fig. 6: Speed measurement as a function of a local constriction in three terminal device. **a.** The scanning electron micrograph image of the three terminal device. This is the same device as in Fig. 4. For the measurement, the gates without false colour including the narrow middle gate are not polarised. The gates coloured in orange are polarised to form a single electron waveguide. The width of the waveguide is kept wide hosting more than 30 conduction channels. The time-resolved measurement is performed by using the gate $\mathrm{QPC_{det}}$. When a relatively small negative voltage is applied on the gate g_{RES}, this device works as a three terminal device. On the other hand, when a large negative voltage is applied on the gate g_{RES} to pinch off the connection to the contact, O_{l}, the device becomes a two terminal device. In this measurement, we use a Gaussian shaped voltage pulse whose full width at half maximum (FWHM) is about 83 ps for both the excitation of plasmon wavepackets and the time-resolved measurement with $\mathrm{QPC_{det}}$. The peak amplitude of the excitation voltage pulse is $\sim 0.5\,\mathrm{mV}$. **b.** Time-resolved measurement of plasmon wavepackets in the three-terminal situation. The different curves are taken at different voltages on the gates g_{QPC}. The amplitude is normalised to one and the different curves are offset vertically for clarity. **c.** Time-resolved measurement as **b** in the two-terminal situation. **d.** Speed of plasmon wavepackets as a function of the voltage on g_{QPC} for the three-terminal situation (blue) and the two-terminal situation (red). The speed of plasmon wavepackets is modified with g_{QPC} only for the two-terminal situation. The number N on top of the grey shaded gate voltage indicates the number of transmitting electron channels across g_{QPC}, which is determined by the observation of the quantised conductance.

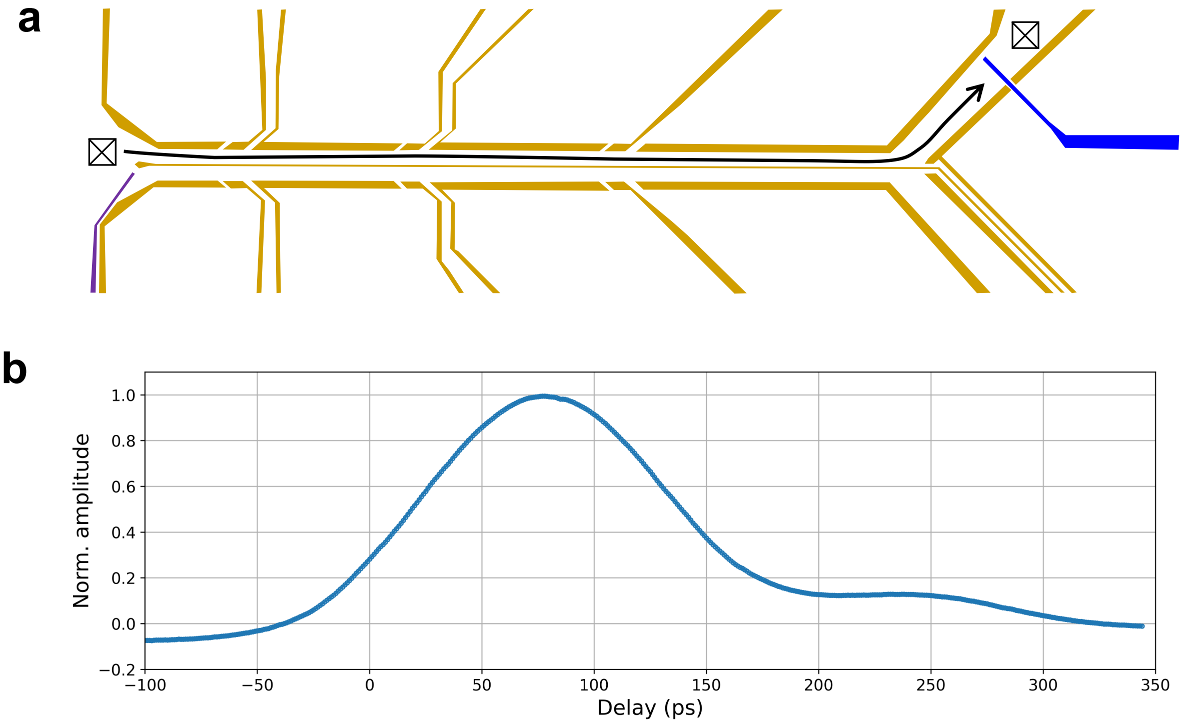

Supplementary Fig. 7: Time-resolved measurement of the injected plasmon wavepacket. a. Schematic showing the device configuration to perform time-resolved measurement of the injected plasmon wavepacket. Here plasmon wavepackets are injected to the upper electron waveguide by closing the lower gate at the entrance highlighted in purple. **b.** Time-resolved measurement of the injected plasmon wavepacket. The observed shape is the convolution of the bare wavepacket shape and opening of the detection gate. Both excitation of the plasmon wavepacket and opening of the detection gate are performed with 83 ps-long voltage pulse from AWG (Keysight M8190A).
